# Neighborhood characteristics and dementia symptomology among community-dwelling older adults with Alzheimer’s disease

**DOI:** 10.3389/fnagi.2022.937915

**Published:** 2022-09-20

**Authors:** Dana M. Alhasan, Matthew C. Lohman, Jana A. Hirsch, Maggi C. Miller, Bo Cai, Chandra L. Jackson

**Affiliations:** ^1^Epidemiology Branch, Department of Health and Human Services, National Institute of Environmental Health Sciences, National Institutes of Health, Research Triangle Park, NC, United States; ^2^Department of Epidemiology and Biostatistics, Arnold School of Public Health, University of South Carolina, Columbia, SC, United States; ^3^Urban Health Collaborative, Department of Epidemiology and Biostatistics, Dornsife School of Public Health, Drexel University, Philadelphia, PA, United States; ^4^Intramural Program, Department of Health and Human Services, National Institute on Minority Health and Health Disparities, National Institutes of Health, Bethesda, MD, United States

**Keywords:** dementia, neuropsychiatric symptoms, behavioral and psychological symptoms, residence characteristics, rural health, poverty area, South Carolina

## Abstract

**Background:**

Neuropsychiatric symptoms (NPSs) lead to myriad poor health outcomes among individuals with Alzheimer’s disease (AD). Prior studies have observed associations between the various aspects of the home environment and NPSs, but macro-level environmental stressors (e.g., neighborhood income) may also disrupt the neuronal microenvironment and exacerbate NPSs. Yet, to our knowledge, no studies have investigated the relationship between the neighborhood environment and NPSs.

**Methods:**

Using 2010 data among older adults with AD collected from a sample of the South Carolina Alzheimer’s Disease Registry, we estimated cross-sectional associations between neighborhood characteristics and NPSs in the overall population and by race/ethnicity. Neighborhood measures (within a 1/2-mile radius of residence) came from the American Community Survey and Rural Urban Commuting Area Code. We categorized median household income into tertiles: < $30,500, $30,500–40,000, and > $40,000, and rurality as: rural, small urban, and large urban. Residential instability was defined as the percent of residents who moved within the past year. NPSs were defined using the Neuropsychiatric Inventory Questionnaire that included the composite measure of all 12 domains. Adjusting for age, sex/gender, race/ethnicity, and caregiver educational attainment, we used negative binomial regression to estimate prevalence ratios (PR) and 95% confidence intervals (CI) for NPSs by neighborhood characteristics.

**Results:**

Among 212 eligible participants, mean age was 82 ± 8.7 years, 72% were women, and 55% non-Hispanic (NH)-Black. Individuals with AD living in < $30,500 vs. > $40,000 income neighborhoods had a 53% (PR = 1.53; 95% CI = 1.06–2.23) higher prevalence of NPSs while individuals living in rural vs. large urban neighborhoods had a 36% lower prevalence of NPSs (PR = 0.64; 95% CI = 0.45–0.90), after adjustment. We did not observe an association between residential instability and NPSs (PR = 0.92; 95% CI = 0.86–1.00); however, our estimates suggested differences by race/ethnicity where NH-White older adults living in residential instable areas had lower NPSs (PR = 0.89; 95% CI = 0.82–0.96) compared to NH-Black older adults (PR = 0.96; 95% CI = 0.86–1.07).

**Discussion:**

Across racial/ethnic groups, individuals with AD had more symptomology when living in lower income areas. Pending replication, intervention efforts should consider resource allocation to high-need neighborhoods (e.g., lower income), and studies should investigate underlying mechanisms for this relationship.

## Introduction

Alzheimer’s disease (AD) is a debilitating neurodegenerative disease that is the sixth leading cause of death in the United States (Murphy et al., 2015; Kochanek et al., 2016). With no known cure for AD and a growing older adult population, the prevalence of AD is projected to substantially increase over the next 40 years (Barnes and Yaffe, 2011). This increase is expected to become disproportionately higher among minoritized groups, including non-Hispanic (NH) Black adults (Matthews et al., 2019). Furthermore, AD is one of society’s costliest conditions with an estimated total direct medical cost of $259 billion in 2017—half of which was covered by Medicare (Alzheimer’s Association, 2017). Managing AD symptoms and delaying progression to late-stage AD could reduce the negative impact on quality of life and wellbeing of individuals with AD as well as costs (Rabins et al., 2013). According to the environmental stress theory, external stressors (e.g., air pollution) found in the environment can substantially affect the neuronal microenvironment and thus the health of individuals and groups (Kagias et al., 2012), including the development and progression of AD (Wainaina et al., 2014). Avoiding or reducing exposure to adverse environmental factors may reduce AD severity and, thus, there is a need to identify environmental, macro-level factors that influence the severity of AD. Such factors are important to develop intervention targets to delay AD severity. Thus, the crux of our study is focused on identifying intervention targets for AD symptomology since primary prevention has been unsuccessful (Rabins et al., 2013), and there is promising evidence that multicomponent interventions may decrease neuropsychiatric symptoms (NPSs) (Livingston et al., 2020).

NPSs of AD are highly correlated with AD severity (Peters et al., 2015). NPSs are considered non-cognitive symptoms that are present during AD and persist throughout disease progression (Steinberg et al., 2004). Common NPSs include apathy, agitation, irritability, delusions, and hallucinations. Clinical studies estimate that 70–90% of AD patients experience at least one NPSs (Steinberg et al., 2004), and a recent systematic review estimated prevalence of NPSs to range from 4 to 32% among community-dwelling patients with AD (Kwon and Lee, 2021). Studies have also identified certain NPSs associated with more AD severity, particularly highlighting apathy (Peters et al., 2015) and agitation (Mulders et al., 2016). Additionally, NPSs, such as apathy, have recently emerged as predictors of disability, faster cognitive decline, and greater mortality (Modrego and Lobo, 2018). For example, apathy has been found to be associated with increased risk of mortality as well as severe cognitive and physical decline (Vilalta-Franch et al., 2013; Spalletta et al., 2015).

While not a marker for AD severity, the consistent association between NPSs and poor health outcomes has led to the widespread acknowledgment of NPSs as a priority research area for AD (Porsteinsson and Antonsdottir, 2017; Halpern et al., 2019). Although the biological basis of NPSs is poorly understood (Ferrari et al., 2018; Hallikainen et al., 2018), NPSs are hypothesized to be influenced by psychological, social, and physical environmental factors (Eriksson, 2000; Modrego and Lobo, 2018). For example, NPSs are thought to be influenced by chronic psychological stress, limited social engagement and mental stimulation, along with exposure to environmental toxicants and stressors (e.g., air pollution, pesticides, and noise) (Lee et al., 2019). Disadvantaged, rural, and residentially unstable neighborhoods may influence NPSs by increasing stress levels, impeding mental stimulation, and lacking opportunities for social engagement, as previously demonstrated with cognitive function (Clarke et al., 2012). Characterized by more environmental toxicants and stressors (Hajat et al., 2013), disadvantaged neighborhoods are theorized to exacerbate NPSs, similar to dementia risk (Killin et al., 2016; Paul et al., 2019) in accordance with the environmental stress concept (Wainaina et al., 2014). Disadvantaged neighborhoods may also influence NPSs by limiting access and quality to resources (e.g., senior centers), health care services, and transportation (Clarke et al., 2012). Level of access is likely also more limited in neighborhoods of predominantly NH-Black residents compared to NH-White residents, which have been historically and systematically under-resourced (Williams and Collins, 2016), and thus it is also important to investigate if differences by race/ethnicity exist. While limited neighborhood resources and insufficient healthcare services may compound and exacerbate NPSs, areas with high neighborhood resources and sufficient healthcare services may alleviate these symptoms (Subramanian et al., 2006). This proposed NPSs-environment relationship among those with AD has been demonstrated in outcomes related to AD severity, such as poor cognition, cognitive decline, and physical impairment (Freedman et al., 2008) but not studied with NPSs. In rural neighborhoods, farther distances to resources and healthcare, coupled with limited transportation methods, may exacerbate NPSs. Moreover, individuals living in rural compared to urban areas have been shown to experience greater social isolation, which is speculated to indirectly speed NPSs development (Nakamura et al., 2016). Caregivers, especially those living in rural areas, are also thought to experience social exclusion (Greenwood et al., 2018), which can indirectly worsen NPSs among individuals with AD *via* their caretaking abilities. NH-Black caregivers and their care recipients may also experience greater levels of social exclusion due to ongoing social disadvantage and discrimination. Given that social isolation and fewer opportunities for social engagement may increase risk of NPSs, residential instability through high turnover of residents may exacerbate NPSs. Residential stability provides opportunities for relationship building, and subsequently is thought to slow NPSs development *via* cognitive stimulation and social support (Rote et al., 2017). Yet, few studies have investigated factors associated with NPSs, especially in terms of neighborhood environment characteristics (Mulders et al., 2016). This is important given the environmental stress concept, which has been demonstrated within interpersonal relationships where, for instance, caregiver stress can influence NPSs among individuals with AD (Kales et al., 2015; Alhasan et al., 2021). Environmental modifications in the home environment, such as light therapy, application of color, or reduction of complexity, have also been demonstrated to decrease NPSs (Eriksson, 2000; Gitlin et al., 2003). Therefore, evaluating whether different neighborhood characteristics are associated with NPSs may help identify potential targets for intervention by which neighborhood environments influence NPSs and potentially AD severity.

Despite the potential links between neighborhood characteristics and NPSs, the association between them, to our knowledge, has not been investigated. In accordance with the environmental stress theory related to AD (Wainaina et al., 2014), this study aimed to understand AD from a contextual, socioecological perspective rather than an individualized one. Our aim was to estimate the association between neighborhood characteristics (i.e., median household income, rurality, and residential instability) and NPSs among community-dwelling older adults with AD living with a caregiver in South Carolina (SC) overall and by race/ethnicity. We hypothesized that older adults with AD living in lower income neighborhoods, more rural areas, and neighborhoods with higher residential instability would be associated with more NPSs. We also hypothesized that these associations would be stronger among NH-Black older adults compared to NH-White older adults.

## Materials and methods

### Study population

Participant data came from a sample collected from the SC AD Registry (Porter et al., 2016). The Registry is a comprehensive statewide roster of diagnosed cases of AD and other related dementias (Miller et al., 2019). In 2010, trained interviewers collected data for the sample by asking caregivers *via* phone about the individuals with AD for whom they cared. The sample inclusion criteria included participants identified from the Registry with an AD diagnosis between 2005 and 2010 (based on International Classification of Diseases 9/10 Clinical Modifications code, which may not have been confirmed with biomarker data), enrolled in a Medicaid waiver program, eligible for nursing home level of care (e.g., can receive additional care services, such as home delivered meals, while still residing within the community), and an informal caregiver available for interview. We excluded participants who did not reside with a caregiver (e.g., participants living in nursing homes) because their addresses were not collected by study staff (*n* = 411; of whom, 283 were community-dwelling AD patients). Therefore, our study is limited to community-dwelling older adults with AD who co-habited with a caregiver. Additional details regarding study data collection and eligibility criteria have been previously published (Porter et al., 2016).

Among the 224 community-dwelling older adults with AD who co-habited with their caregiver, we excluded 12 cases because their residence (e.g., P.O. Box) could not be verified. We geocoded the remaining 212 observations using ArcGIS Desktop Version 10.2.2 for Windows (Environmental Systems Research Institute, Redlands, CA) (Supplementary Figure 1). We compared participants included in our study to the full participant sample and found no substantial differences due to our exclusions (Supplementary Table 1). The study obtained verbal informed consent from all participants before the study (Porter et al., 2016), and this study was deemed exempt by the Institutional Review Board at the University of South Carolina (ID = Pro00076582) and by the National Institute of Environmental Health Sciences’ Institutional Review Board because it was considered secondary data.

### Exposure assessments: Neighborhood characteristics

We obtained neighborhood characteristic data at the census tract level from two secondary online sources: the 2006–2010 American Community Survey (ACS) District of Columbia (2011) and the 2010 US Department of Agriculture Rural-Urban Commuting Area (RUCA) codes District of Columbia (2013). We obtained shapefiles and geographic features for SC from the US Census Topologically Integrated Geographic Encoding and Referencing (TIGER) Line Files (Bryson, 2010).

We defined neighborhoods as 1/2-mile Euclidean (or radial) buffer distances around each participants’ geocoded address. Because individual residence-based buffers tend to overlap multiple census tracts, we calculated neighborhood characteristics as the area-weighted average of proportion of intersecting census tracts within the buffer. We chose this smaller buffer size, compared to other sizes in the field (e.g., 1, 3, and 5-mile) because individuals with AD tend to be less mobile and do not travel far away from their residence. This buffer size has also been previously used in research among older adults (Besser et al., 2019). Neighborhood characteristics included residential instability, defined as the percent of neighborhood residents who moved within the past year, and median annual household income categorized into tertiles: < $30,500, $30,500–40,000, and > $40,000.

We assigned participant rurality by the census tract in which the geocoded addresses resided. RUCA measures rurality based on population density, urbanization, and daily commuting on a 10-point scale ranging from metropolitan to rural. We divided census tracts into three rurality categories: (1) large urban (1 = metropolitan area core; *n* = 105), (2) small urban (2 = metropolitan area high commuting and 3 = metropolitan area low commuting; *n* = 41), and (3) rural (4 = micropolitan area core, 5 = micropolitan high commuting, 6 = micropolitan low commuting, 7 = small town core, 8 = small town high commuting, 9 = small town low commuting, and 10 = rural areas; *n* = 66).

### Outcome assessment: Neuropsychiatric symptoms

We measured NPSs using the Neuropsychiatric Inventory Questionnaire (NPI-Q), an abbreviated, validated version of the original survey (Kaufer, 2000). The NPI-Q consists of 12 domains: delusions; hallucinations; agitation/aggression; depression/dysphoria; anxiety; elation/euphoria; apathy/indifference; disinhibition; irritability/lability; motor disturbance; sleep, and nighttime behavior disorders; and appetite/eating changes. Within each of these 12 domains, a respondent caregiver is asked if these symptoms are present or absent. For the symptoms that are present, the caregiver is asked to rate both the severity on a 3-point scale (1 = mild, 2 = moderate, and 3 = severe) and the frequency on a 4-point scale (1 = occasionally, 2 = often, 3 = frequently, and 4 = very frequently). Multiplying the severity and frequency scores for each symptom produces a domain score. The domain scores, when summed across all 12 domains, yield a composite NPSs score with lower scores indicating less NPSs (most favorable) and higher scores indicating more NPSs (least favorable). NPSs score was considered as count data (analyzed continuously) and ranges from 0 to 95.

### Potential confounders

Confounders were selected *a priori* using a directed acyclic graph (DAG): current age (measured continuously), sex/gender (men or women), self-identified race/ethnicity (NH-Black or other including NH-White, Hispanic/Latinx, and Asian), and caregiver educational attainment [< 8th grade, 8th–12th grade, ≥ high school, and unknown (8.4%)].

### Potential effect modifier

We also considered self-identified race/ethnicity (NH-Black or NH-White) as a potential effect modifier given known differences in neighborhood characteristics (e.g., income) by race/ethnicity due to, for instance, historical and contemporary forms of structural racism (Bailey et al., 2017) resulting in racial residential segregation (Williams and Collins, 2016). Also, there are known differences in AD risk and subsequent NPSs by race/ethnicity (Matthews et al., 2019).

### Statistical analyses

We computed descriptive statistics and presented categorical data as numbers with percentages and continuous data as means with standard deviations (SDs). To test differences between NPSs (i.e., NPI domains) among NH-White and NH-Black older adults, we used *t*-tests. We used negative binomial regression models to estimate prevalence ratios (PRs) with 95% confidence intervals (CIs) between neighborhood characteristics (< $30,500 and $30,500–40,000 vs. > $40,000, rural and small urban vs. large urban, and one percent increase in residents who moved) and NPSs score. Unlike most studies that treat NPI as normally distributed, we accounted for the non-normal distribution of NPI scores using non-parametric statistics. We included the following confounders in the overall statistical model: age, sex/gender, race/ethnicity, and caregiver educational attainment. We assessed variable diagnostics using the Pearson chi-square test of deviance. We set the significance level at 0.05 and completed all analyses using SAS software, Version 9.3 for Windows (SAS Institute, Cary, NC).

### Secondary analyses

Because empirical evidence suggests certain NPSs (i.e., apathy, agitation, and irritability) are related to more NPSs and, in general, higher AD severity (9, 11), we employed negative binomial regressions to assess separate associations between neighborhood characteristics and three NPSs (apathy, agitation, and irritability) overall (Supplementary Table 2) and by race/ethnicity (Supplementary Table 3). To account for uncertainty in relevant spatial scale, we conducted a sensitivity analysis using a 1-mile buffer distance. This resulted in no noted significant differences between the models using different neighborhood definitions (1/2- vs. 1-mile), and, therefore, we present the 1/2-mile buffer results.

## Results

### Study population characteristics

Among 212 community-dwelling older adults with AD and living with a caregiver, mean age was 82.4 years (*S.D.* = 8.7), the women:men ratio was 2.5:1, and over half of the study population were NH-Black (55.1%) (Table 1). A higher percent of NH-White older adults caregivers had a high school education or more (46.2%) compared to NH-Black older adults caregivers (19.6%). Almost half of the participants lived in large urban (49.5%) and average median household income ($37,485) neighborhoods. A higher percentage of NH-Black older adults compared to NH-White older adults lived in rural (33.3% vs. 29.0%) and < $30,500 income (37.6% vs. 26.1%) neighborhoods. Participants lived in neighborhoods where on average 3.8% of residents moved the past year (Table 1). The total NPSs score had a mean of 26.3 (range = 0–95; *S.D.* = 22.3) (Table 2). Domains with the highest means were agitation, irritability, apathy, and motor disturbances; euphoria was the domain with the lowest mean. NH-White older adults had a higher mean of total NPSs score (30.6; *S.D.* = 22.7) compared to NH-Black older adults (23.2; *S.D.* = 21.7). NH-White older adults also had higher means in almost all NPI domains compared to NH-Black older adults (Table 2).

**TABLE 1:** Demographics and neighborhood characteristics of community-dwelling older adults with Alzheimer’s disease living with a caregiver and stratified by race/ethnicity, 2010 (*n* = 212).

	Overall *N* = 212 (100%)	NH-black *N* = 117 (55%)	NH-white *N* = 93 (45%)
Demographics	*N* (%)	*N* (%)	*N* (%)
**Age**, mean ± S.D., years	82.4 ± 8.7	82.2 ± 8.4	82.6 ± 9.1
**Sex/gender**			
Men	58 (27.3)	35 (29.9)	22 (23.6)
Women	154 (72.7)	82 (70.1)	71 (76.4)
**Race/ethnicity** ^a^			
Non-Hispanic black	117 (55.1)	–	–
Non-Hispanic white	93 (43.9)	–	–
**Caregiver educational attainment**			
<8th grade	75 (35.3)	49 (41.8)	26 (27.9)
8th–12th grade	53 (25.0)	31 (26.5)	21 (22.6)
≥High school^b^	66 (31.3)	23 (19.6)	43 (46.2)
Unknown/refused	18 (8.4)	14 (11.9)	3 (3.2)

**Neighborhood characteristics**	***N*** **(%)**	***N*** **(%)**	***N*** **(%)**

**Rurality** ^c^			
Large urban	105 (49.5)	51 (43.5)	53 (56.9)
Small urban	41 (19.3)	27 (23.1)	13 (13.9)
Rural	66 (31.1)	39 (33.3)	27 (29.0)
**Median household income**, mean ± S.D.	$37,485.20 ± 12,867.80	$36,368.60 ± 12,590.20	$38,904.00 ± 13,268.60
>$40,000	71 (33.4)	34 (29.1)	37 (39.8)
30,500–40,000	73 (34.4)	39 (33.3)	31 (33.1)
<$30,500	68 (32.2)	44 (37.6)	25 (26.1)
**Residential instability**^d^, mean ± S.D.	3.8 ± 2.1	3.7 ± 2.1	4.0 ± 2.1

^a^Other race/ethnicity included Hispanic/Latinx (*n* = 1) and Asian (*n* = 1) participants who were excluded due to low sample size. ^b^Caregiver educational attainment high school and more included individuals who completed the General Education Development (*n* = 46), some college (*n* = 15), and graduated college (*n* = 5). ^c^Rurality was measured based on the RUCA (Rural Urban Commuting Area codes) where large urban was defined as metropolitan area core; small urban was defined as metropolitan area high commuting and metropolitan area low commuting; and rural was defined as micropolitan area core, micropolitan high commuting, micropolitan low commuting, small town core, small town high commuting, small town low commuting, and rural areas. ^d^Residential instability was defined as the percent of residents who moved the past year and measured continuously.

**TABLE 2:** Mean scores of neuropsychiatric inventory questionnaire a domains in community-dwelling older adults with Alzheimer’s disease living with a caregiver and stratified by race/ethnicity, 2010 (*n* = 212).

	Overall *N* = 212 (100%)	NH-black^a^ *N* = 117 (55%)	NH-white^b^ *N* = 93 (45%)	*P*-value
NPI domain	Mean ± S.D.^c^	Mean ± S.D.	Mean ± S.D.	
NPI total score	26.3 ± 22.3	23.2 ± 21.7	30.6 ± 22.7	0.0176
Delusions	1.8 ± 3.1	1.7 ± 3.1	1.9 ± 3.2	0.6810
Hallucinations	1.9 ± 3.1	2.0 ± 3.2	1.8 ± 2.8	0.6851
Agitation/aggression	3.1 ± 3.5	2.8 ± 3.4	3.6 ± 3.8	0.0883
Depression/dysphoria	2.3 ± 3.5	1.8 ± 3.0	3.0 ± 4.0	0.0149
Anxiety	1.7 ± 3.1	1.3 ± 2.7	2.3 ± 3.5	0.0170
Euphoria/elation	0.7 ± 1.7	0.9 ± 2.0	0.7 ± 1.5	0.1151
Apathy	2.8 ± 3.7	2.4 ± 3.6	3.4 ± 3.9	0.2937
Disinhibition	1.3 ± 2.7	1.1 ± 2.1	1.8 ± 3.3	0.0584
Irritability	2.8 ± 3.7	2.6 ± 3.6	3.3 ± 3.9	0.1723
Motor disturbances	2.8 ± 3.7	2.3 ± 3.6	3.5 ± 4.0	0.0209
Sleep and nighttime disturbances	2.7 ± 3.8	2.6 ± 3.6	3.0 ± 4.3	0.4972
Appetite/eating change	1.9 ± 3.3	1.7 ± 3.1	2.3 ± 3.6	0.2422

^a^The Neuropsychiatric Inventory Questionnaire (NPI-Q) assesses neuropsychiatric symptoms and ranges from 0 to 95. Higher scores indicate more neuropsychiatric symptoms, and lower scores indicate less neuropsychiatric symptoms. ^b^Other race/ethnicity included Hispanic/Latinx (*n* = 1) and Asian (*n* = 1) participants who were excluded due to low sample size. ^c^S.D., standard deviation.

### Neighborhood characteristics and neuropsychiatric symptoms

We estimated that participants who live in small urban areas have 31% (PR = 0.69; 95% CI = 0.48–0.98) prevalence of lower NPSs vs. urban neighborhoods and rural areas have a prevalence 36% lower (PR = 0.64; 95% CI = 0.45–0.90), after adjustment (Table 3). We estimated that participants who live in < $30,500 income neighborhoods have 1.53 (95% CI = 1.06–2.23) and $30,500–40,000 income neighborhoods have 1.21 times (95% CI = 0.86–1.69) as high NPSs vs. > $40,000 income neighborhoods, after adjustment. Moreover, a one percent increase in the proportion of residents who moved within the past year resulted in an 8% decrease (PR = 0.92; 95% CI = 0.86–1.00) in the average NPSs score after adjustment, although this failed to reach statistical significance (Table 3). However, our estimates differed by race/ethnicity where NH-White older adults living in residentially instable areas had lower NPSs compared to NH-Black older adults (PR_NH–White_ = 0.89; 95% CI = 0.82–0.96; PR_NH–Black_ = 0.96; 95% CI = 0.86–1.07) (Table 4). There were no other differences observed between neighborhood income as well as rurality by race/ethnicity.

**TABLE 3:** Prevalence ratios of total neuropsychiatric symptoms by neighborhood characteristics, 2010 (*n* = 212).

	Unadjusted PR (95% CI)	Adjusted^c^ PR (95% CI)
**Rurality** ^a^		
Rural	0.81 (0.60–1.08)	**0.64 (0.45**–0.90)**
Small urban	0.78 (0.55–1.10)	0.69 (0.48–0.98)*
Large urban	1.00^d^	1.00^d^
**Median household income**		
<$30,500	1.13 (0.82–1.55)	**1.53 (1.06**–2.23)*
30,500–40,000	0.98 (0.72–1.34)	1.21 (0.86–1.69)
>$40,000	1.00^d^	1.00^d^
**Residential instability** ^b^	0.94 (0.88–1.00)	0.92 (0.86–1.00)*

^a^Rurality was measured based on the RUCA (Rural Urban Commuting Area codes) where large urban was defined as metropolitan area core; small urban was defined as metropolitan area high commuting and metropolitan area low commuting; and rural was defined as micropolitan area core, micropolitan high commuting, micropolitan low commuting, small town core, small town high commuting, small town low commuting, and rural areas. ^b^Residential instability was defined as the percent who moved the past year and measured continuously. ^c^Model was adjusted for individual Alzheimer’s Disease patient age, sex/gender, race/ethnicity, and caregiver educational attainment. ^d^Reference category. *p < 0.05; **p < 0.01. Bolded estimates indicate statistical significance.

**TABLE 4:** Prevalence ratios of total neuropsychiatric symptoms by neighborhood characteristics among non-Hispanic black and non-Hispanic white older adults, 2010.

	NH-black adults with AD (*n* = 117)		NH-white adults with AD (*n* = 93)	
	Unadjusted PR (95% CI)	Adjusted^c^ PR (95% CI)	Unadjusted PR (95% CI)	Adjusted^c^ PR (95% CI)
**Rurality** ^a^				
Rural	0.78 (0.51–1.21)	0.62 (0.37–1.04)	0.87 (0.60–1.28)	0.69 (0.45–1.05)
Small urban	0.89 (0.55–1.46)	0.84 (0.49–1.45)	0.68 (0.42–1.11)	0.65 (0.41–1.04)
Large urban	1.00^d^	1.00^d^	1.00^d^	1.00^d^
**Median household income**				
<$30,500	1.31 (0.82–2.10)	1.72 (0.98–3.02)	1.04 (0.69–1.58)	1.48 (0.94–2.34)
30,500–40,000	1.22 (0.75–1.97)	1.43 (0.86–2.36)	0.88 (0.59–1.30)	1.14 (0.75–1.73)
>$40,000	1.00^d^	1.00^d^	1.00^d^	1.00^d^
**Residential instability** ^b^	0.97 (0.88–1.07)	0.96 (0.86–1.07)	**0.90 (0.83**–0.97)**	**0.89 (0.82**–0.96)**

^a^Rurality was measured based on the RUCA (Rural Urban Commuting Area codes) where large urban was defined as metropolitan area core; small urban was defined as metropolitan area high commuting and metropolitan area low commuting; and rural was defined as micropolitan area core, micropolitan high commuting, micropolitan low commuting, small town core, small town high commuting, small town low commuting, and rural areas. ^b^Residential instability was defined as the percent who moved the past year and measured continuously. ^c^Model was adjusted for individual Alzheimer’s Disease patient age, sex/gender, and caregiver educational attainment. ^d^Reference category. **p < 0.01. Bolded estimates indicate statistical significance.

## Discussion

Among community-dwelling older adults with AD who were also living with a caregiver, we found a hypothesis-supporting association suggesting that individuals living in low vs. high-income neighborhoods experienced more NPSs. However, our study results did not support the hypothesis that residing in rural vs. urban neighborhoods is associated with more NPSs. We also did not find evidence in support of the hypothesis that those with greater than average NPSs lived in areas with higher residential instability among the overall population and NH-Black adults. In contrast, we found that living in areas with higher residential instability was associated with lower NPSs only among NH-White older adults, which did not align with our hypothesis.

While myriad studies have evaluated individual-level factors associated with NPSs (Modrego and Lobo, 2018), few have investigated the factors specifically among individuals with AD. Therefore, our study extends prior research. We observed a mean NPS score that was similar to average scores found in previous studies also among community-dwelling older adults with AD (Aalten et al., 2005; Srikanth et al., 2005) or a slightly higher NPS score compared to others. In particular, our observed mean is higher compared to the Utah-based Cache County Dementia Progression Study, which reported a mean of 8.9 (*S.D.* = 14.30) among 214 AD patients (Lyketsos et al., 2000), and another study based in Finland that reported a mean of 8.89 (*S.D.* = 9.69) among 236 very mild and mild AD patients (Hallikainen et al., 2018). Given the higher prevalence of AD in SC (which had the highest AD-related mortality rate in 2018) (Miller et al., 2019), this observation was not surprising. Further, this observation was expected since our sample included those eligible for nursing home level of care, where institutionalization is associated with more NPSs (Li et al., 2014). Finally, our study reports NPSs by race/ethnicity, which is rarely available in other work.

While no other studies, to our knowledge, have focused only on NPSs, our findings that individuals with AD living in lower income neighborhoods experienced more NPSs are aligned with previous literature focusing on the neighborhood environment, including neighborhood income, and cognition (Besser et al., 2017). A similar study focusing on dementia incidence also found that adults living in socioeconomically disadvantaged neighborhoods were at greater risk for dementia, independent of age, sex/gender, and education (Berr et al., 2015). Previous studies of cognitively healthy individuals found similar results regarding the relationship between more neighborhood disadvantage and more physical impairment or cognitive decline, two factors also correlated with AD severity (Balfour and Kaplan, 2002; Clarke and George, 2005; Freedman et al., 2008; Beard et al., 2009; Clarke et al., 2015). Lower income neighborhoods may influence NPSs- and ultimately AD severity- through chronic psychological stress. Air pollution, for example, leads to reactive oxygen species production, which is a biochemical reaction that occurs during the processes of respiration and increases AD risk (Block and Calderon-Garciduenas, 2009). Social isolation is associated with abnormalities in long-term potentiation and dendritic branching, as shown in animal studies, and may therefore negatively impact contextual memory (Kamal et al., 2014). Other chronic stressors, like psychosocial stress, lead to impaired adult neurogenesis in the hippocampus, as shown in animal studies, and increase AD risk (Thomas et al., 2007). In particular, chronic stressors may contribute to racial disparities where one study reported older Black adults with higher vs. lower levels of perceived stress had more rapid decline in cognitive function (Turner et al., 2017). Black older adults who attended desegregated schools in the South, which is associated with higher psychosocial stress, had poorer cognition than those born in the North (Lamar et al., 2020). Because previous studies have demonstrated the role of these factors with poor cognition and risk of AD (Moulton and Yang, 2012; Wainaina et al., 2014), future research can determine more precisely what components of neighborhood economic disadvantage matter most for NPSs.

Our finding that older adults living in rural vs. urban neighborhoods experienced less NPSs did not align with our hypothesis. Rural neighborhoods are characterized by fewer resources, less access to care, and more social isolation, all of which have the potential to increase NPSs, as previously shown with cognitive impairment (Nakamura et al., 2016) and dementia (Russ et al., 2012). Nonetheless, because rural neighborhoods have fewer environmental stressors compared to urban neighborhoods, it is possible that the quiet, serene, and naturalistic settings found in rural neighborhoods are a potential explanation for our observation of lower NPSs among individuals living in rural compared to urban neighborhoods (Verheij et al., 2008). Some studies have found that stressors common in urban areas, such as excessive noise, are associated with more dementia, cardiovascular disease, and stroke (Paul et al., 2019) and may be associated with NPSs, especially specific symptoms such as agitation (Kales et al., 2015). As postulated in the environmental stress theory (Kagias et al., 2012), some researchers have theorized that adults with AD may also be more sensitive to these external stressors (Gitlin et al., 2003). Because individuals with AD have progressive difficulty processing and responding to environmental stimuli, excessive noise can lower the biological stress threshold and increase potential for higher levels of frustration (Gitlin et al., 2003). Furthermore, rural neighborhoods have less traffic and street integration (e.g., less turns required to be made from a street segment to reach all other street segments in a defined area) compared to urban neighborhoods, which can make rural areas easier to navigate among individuals with AD and thus potentially be associated with lower NPSs. Watts et al. (2015), found that high neighborhood integration (a measure of number of turns required to travel between two points) was associated with a larger decline in attention over a 2-year period among adults with mild AD. There are more complex cognitive abilities required to navigate a neighborhood, which can discourage older adults with AD from venturing and walking. In fact Brorsson et al. (2016), found that moving around in a complex and dynamic environment is exhausting for individuals with dementia and cognitive limitations. Another potential explanation for these findings is that individuals with more NPSs and have severe AD tend to gravitate to urban neighborhoods to access care and resources that may help caregivers deal with troublesome behavioral and psychiatric issues. Given that the average diagnosis period is somewhat recent for our sample (mid-point of 2007), this is an unlikely explanation. There is also a possibility of reporting bias, where the role of caregiving may be viewed differently by racial/ethnic groups and thus influence how caregivers report NPSs among individuals with AD. Specifically, NH-Black caregivers, who make up 60% of the sample living in rural areas, may have different expectations and perceptions on caregiving compared to NH-White caregivers (e.g., filial responsibility beliefs, religion, and collectivism) (Dilworth-Anderson et al., 2002; Pinquart and Sorensen, 2005). While there is limited information in the literature regarding NPSs differences by race/ethnicity, one study found that NH-Black caregivers may be more likely to underreport NPSs (Cothran et al., 2015).

The null findings regarding residential instability and NPSs differ from the literature examining psychotic symptoms among children (Solmi et al., 2020) and the literature examining physical disability among older adults without dementia (Beard et al., 2009; Nguyen et al., 2016). For instance, a longitudinal study in the UK found greater neighborhood social fragmentation at birth (comprised of four measures including the percent of individuals in a household who moved within the last year) to be associated with more negative symptoms, such as apathy, in adolescence (OR = 1.43; 95% CI = 1.06–1.85) after individual and maternal level adjustment (Solmi et al., 2020). Furthermore, Beard et al. (2009) found residential instability to be associated with a higher prevalence of physical disability, a factor correlated with AD severity like NPSs. In a similar manner, Nguyen et al. (2016) found that adults with a mean age of 65.7 years and who lived in neighborhoods with high social cohesion (measured *via* self-report of feelings of trust, feeling part of the area, and feeling that people are friendly or would help them if they were in trouble) experienced fewer limitations in activities of daily living that allow an individual to live independently after 8 years. A potential explanation may be that our sample consists of individuals who are eligible for nursing home level of care and thus have severe AD, where the hindrance of residentially instable, social incohesive neighborhoods may be unrelated to exacerbating NPSs. Further, the low variability in the percentage of residents who had moved within the past year may be too small to capture differences in residentially instable areas that might be associated with NPSs, especially considering the small neighborhood definition. Studies have more commonly used percentages of residents who lived in the same house over the past 5 years to capture stability (Hybels et al., 2006; Beard et al., 2009); however, this information was not available in the ACS for our study’s timeframe.

Although our findings suggest NH-White older adults living in residentially instable areas experienced on average lower NPSs, these results may be due to differences in how caregivers report NPSs, as previously mentioned. Given that the average of NPSs is higher among NH-White adults than NH-Black adults, this is a likely explanation. Nonetheless, future research should explore differences by race/ethnicity among a larger sample size.

Our cross-sectional study only provides a snapshot of the relationship between neighborhood characteristics and NPSs. Because we did not have geographic data on whether individuals with AD have moved residence since their diagnosis, we were unable to assess how changes in neighborhood environments may have impacted their NPSs, which is an important pursuit for future research. Moving residence after an AD diagnosis is common, especially to access healthcare services. It is likely that many individuals in this study moved residence, as they were living with a caregiver during the time of data collection. Yet, because the NPI-Q only asks about NPSs over the past month, this timeframe limits the possibility of our results being heavily impacted by changes in the neighborhood environment and thus we are more interested in contemporary rather than historical neighborhood characteristics. Further, studies show that NPSs persist throughout disease progression (Vilalta-Franch et al., 2013; Kales et al., 2015). Another limitation is that there may be selection bias regarding which caregivers chose to participate in the study, in that caregivers with a recipient with more AD severity might be less likely to respond as they are providing continuous care. Given no data regarding non-response or AD severity, we were unable to conduct a sub-analysis to see how this impacts our study; however, the initial response rate from the original collected sample was relatively high (72%) (Porter et al., 2016). Similarly, our sample consists of participants enrolled in a Medicaid waiver program, which can limit the generalizability of the results to individuals with low income. The use of caregiver reported data may also introduce misclassification by race/ethnicity regarding differences in reporting NPSs. A fifth limitation to note is the lack of individual-level variables, such as individual socioeconomic status (SES), which can potentially mediate the relationship between low-income neighborhoods and NPSs. Because low-income neighborhoods can operate as a compositional variable *via* proxy for individual-level SES, low-income neighborhoods expose individuals to a cluster of risk factors (e.g., low wealth) resulting in increased exposure to stressors and decreased social and physical resources (Rosso et al., 2016). Although individual income and educational attainment was not available in our dataset to explore this relationship further, we used caregiver educational attainment in our model, which has been previously shown to be similar to the care recipients’ education level (Nichols et al., 2011) or slightly higher than the recipients’ education level (Covinsky et al., 2003; Tanner et al., 2015). Similarly, other important confounders to consider (e.g., physical disability) were not available in the sample and thus may result in unmeasured confounding. We also do not have a measure of how much participants, if at all, access their neighborhood (e.g., life space). A final limitation to note is not considering multiple social categories (e.g., sex/gender; age) that may interact at the individual-level with the neighborhood environment as a result of macro-level systems (Bowleg, 2012). While we examined differences by race/ethnicity (albeit limited to NH-Black and NH-White adults), we did not incorporate further stratification due to our small sample size (*n* = 212) and lack of data on other social categories (e.g., sexual orientation).

Despite the study limitations, there are also important strengths. For instance, research on neighborhood environments and NPSs among the overall population and by race/ethnicity is sparse, and it is important to study racial/ethnic disparities given the disproportionate impact of AD among minoritized racial/ethnic groups. Moreover, we collected and analyzed data from the NPI-Q, which is a highly validated and reliable questionnaire (Kaufer, 2000). The NPI-Q is comprehensive, avoids symptom overlap, and is easy to use (Lai, 2014). The designer of the questionnaire intended for caregivers to complete the survey responses, as they are generally the most appropriate people to report behaviors. This is based on the rationale that older adults with AD are often unable to recall or describe their symptoms (Cummings et al., 1994). Given the gaps presented in NPI distribution (i.e., non-normally distributed), researchers have cautioned against using parametric methods in analysis (Perrault et al., 2000; Lai, 2014) and instead, recommended the use of non-parametric statistics when assessing NPI (Perrault et al., 2000), as we have done. Another strength of our research was the definition of the study participant’s neighborhood at a small spatial scale, given that individuals with AD are less likely to interact with their broader environment compared to healthy older adults. This same neighborhood definition has also been previously used (Besser et al., 2019). Neighborhood income and residential instability, although defined using administrative data, were not defined at the administrative boundary level (e.g., census tracts) but instead at the buffer zone; however, rurality was only available at the census tract level. Finally, although our observations were derived from data obtained over a decade ago, they are still relevant today, as the average median household income in South Carolina in 2020 remains low despite inflation ($54,864) (U.S. Census Bureau, 2020).

NPSs are strongly associated with psychological stress and depression in caregivers, as well as with reduced income from employment and lower quality of life (Kales et al., 2015). Because our sample population of community-dwelling older adults with AD was limited to those who lived with caregivers, a living situation which may impact both NPSs and AD severity, future research should examine the association between neighborhood environments and AD severity among community-dwelling older adults who do not live with a caregiver.

Our study provides insight into the association between neighborhood characteristics or features of the contextual environment as one potential avenue to influence and, therefore, combat AD severity (Borsje et al., 2014). Overall, we observed that older adults living in lower income and highly populated urban neighborhoods had more severe NPSs. Because the underlying biological mechanisms of NPSs remain unknown (Ferrari et al., 2018), more research is needed to uncover the underlying mechanisms between neighborhood environments and NPSs. For example, future research can assess if the lower income neighborhood-more NPSs relationship is specifically due to lower access to care, traffic, air pollution, pesticide exposure, low-walkability, low social cohesion, or other potential mechanisms. Identifying neighborhood environments with or at risk of high NPSs is important to delay the progression of AD. NPSs are among the most complex, stressful, and costly aspects of care, and they lead to myriad poor health outcomes, including excess morbidity, mortality, and loss of independence due to early placement in nursing homes (Kales et al., 2015). In cases where NPSs (e.g., apathy) persist, there will be continued higher risk of institutionalization, comorbidities, and mortality (Modrego and Lobo, 2018). This supports an approach to identify macro-level characteristics among low-income that influence NPSs and AD severity to offer targets for intervention that can slow or diminish AD-associated morbidity. As there is significant, yet limited, progress in the pharmaceutical industry, we encourage researchers to look for a more comprehensive array of more viable, cost-effective treatment options (Qiu et al., 2017). As a result, provision of resources to individuals with AD and high-need, resource-poor neighborhoods can increase access to more accessible treatments and access to care. Eventually, policies can be implemented that support dementia-friendly neighborhoods in a manner that helps engender a society where individuals with AD and other forms of dementia can continue to engage in everyday activities with an optimal quality of life—free from NPSs (Innes, 2013).

## Data availability statement

The original contributions presented in this study are included in the article/Supplementary material, further inquiries can be directed to the corresponding author/s.

## Ethics statement

The studies involving human participants were reviewed and approved by the Institutional Review Board at the University of South Carolina (ID: Pro00076582). Written informed consent for participation was not required for this study in accordance with the national legislation and the institutional requirements.

## Author contributions

DA, ML, and CJ designed the study. DA and MM acquiesced the data. DA and BC completed the analysis. DA drafted the manuscript. JH and CJ provided administrative, technical, and material support as well as funding. All authors critically revised the manuscript.
