# Supplementary material for: Neighborhood characteristics and dementia symptomology among community-dwelling older adults with Alzheimer’s disease
Source: Front Aging Neurosci. 2022 Sep 20;14:937915. doi: 10.3389/fnagi.2022.937915 (PMC9530440; doi:10.3389/fnagi.2022.937915)
Supplement: Supplementary file 1 [file Data_Sheet_1.docx]

Supplementary Material

# Supplemental Table 1. Comparing Community-Dwelling Older Adults with Alzheimer’s Disease Living with a Caregiver Demographics Among those in Study Sample vs. Full Sample, 2010

| **Care Recipient Demographics** | **Co-Habited AD Patients from Study Sample (n=212)** | **All Co-Habited AD Patients from Registry Subsample (n=283)** |
| --- | --- | --- |
|  | **N (%)** | **N (%)** |
| NPI ^a^ total score, mean (S.D.) | 26.3±22.3 | 27.1±22.6 |
| Age, mean (S.D.) | 82.4±8.7 | 82.7±8.6 |
| Sex/Gender |  |  |
| Men | 58 (27.4) | 77 (27.2) |
| Women | 154 (72.6) | 206 (72.78) |
| Race/Ethnicity |  |  |
| Non-Hispanic Black | 117 (55.1) | 158 (55.8) |
| Other ^b^ | 95 (44.9) | 125 (44.2) |
| Caregiver Educational Attainment |  |  |
| <8^th^ grade | 75 (35.4) | 109 (38.5) |
| 8^th^ – 12^th^ grade | 53 (25.0) | 64 (22.6) |
| ≥High School ^c^ | 66 (31.3) | 82 (28.9) |
| Unknown/Refused | 18 (8.5) | 28 (9.9) |

^a^ Neuropsychiatric Inventory (NPI) Questionnaire assesses neuropsychiatric symptoms. Higher scores indicate more symptoms and lower scores indicate less symptoms.

^b^ Other race/ethnicity included non-Hispanic white (n=87), Hispanic/Latinx (n=1), and Asian (n=1).

^c^ Caregiver educational attainment high school and more included those who completed the GED (n=46), some college (n=15), and graduated college (n=5).

# Supplemental Table 2. Prevalence Ratios of Apathy, Agitation, and Irritability by Neighborhood Characteristics, 2010 (n=212)

| **Variable** | **Unadjusted PR (95% CI)** | **Adjusted** ^c^ **PR (95% CI)** |
| --- | --- | --- |
| Apathy | | |
| Rurality ^a^ |  |  |
| Rural | 0.79 (0.46-1.36) | 0.69 (0.36 - 1.30) |
| Small Urban | 0.55 (0.29-1.05) | **0.47 (0.23 – 0.94) ^*^** |
| Large Urban | 1.00 ^d^ | 1.00 ^d^ |
| Median household income |  |  |
| Low (<$30,500) | 0.92 (0.51-1.64) | 1.25 (0.63 – 2.50) |
| Medium ($30,500-40) | 0.72 (0.40-1.28) | 0.87 (0.46 - 1.65) |
| High (>$40,000) | 1.00 ^d^ | 1.00 ^d^ |
| Residential Instability ^b^ | 0.95 (0.85-1.06) | 0.93 (0.82 - 1.05) |
| Agitation | | |
| Rurality ^a^ |  |  |
| Rural | 0.75 (0.49-1.14) | **0.62 (0.38 – 1.01) ^*^** |
| Small Urban | 0.62 (0.38-1.03) | **0.60 (0.36 - 1.02) ^*^** |
| Large Urban | 1.00 ^d^ | 1.00 ^d^ |
| Median household income |  |  |
| Low (<$30,500) | 1.12 (0.71-1.76) | 1.53 (0.92 - 2.56) |
| Medium ($30,500-40) | 0.88 (0.56-1.38) | 1.20 (0.73 – 1.97) |
| High (>$40,000) | 1.00 ^d^ | 1.00 ^d^ |
| Residential Instability ^b^ | 0.84 (0.85-1.03) | 0.92 (0.83 - 1.01) |
| Irritability | | |
| Rurality ^a^ |  |  |
| Rural | 0.88 (0.54-1.44) | **0.51 (0.29 - 0.89) ^**^** |
| Small Urban | 0.40 (0.22-0.74) | **0.29 (0.15 - 0.55) ^**^** |
| Large Urban | 1.00 ^d^ | 1.00 ^d^ |
| Median household income |  |  |
| Low (<$30,500) | 1.56 (0.91-2.30) | **2.37 (1.30 - 4.33) ^**^** |
| Medium ($30,500-40) | 1.05 (0.61-1.80) | 1.57 (0.89 – 2.77) |
| High (>$40,000) | 1.00 ^d^ | 1.00 ^d^ |
| Residential Instability ^b^ | 0.98 (0.88-1.10) | 0.94 (0.83 - 1.04) |

^a^ Rurality was measured based on the RUCA (Rural Urban Commuting Area codes) where large urban was defined as metropolitan area core; small urban was defined as metropolitan area high commuting and metropolitan area low commuting; and rural was defined as micropolitan area core, micropolitan high commuting, micropolitan low commuting, small town core, small town high commuting, small town low commuting, and rural areas.

^b^ Residential instability was defined as the percent who moved the past year.

^c^ Model was adjusted for individual AD patient age, sex/gender, race/ethnicity, and caregiver educational attainment.

^d^ Reference category.

^*^p<.05

Bolded estimates indicate statistical significance.

# Supplemental Table 3. Prevalence Ratios of Apathy, Agitation, and Irritability by Neighborhood Characteristics Stratified by Race/Ethnicity, 2010

| **Variable** | **Black adults with AD (n=117)** | | **Other adults with AD (n=95) ^c^** | |
| --- | --- | --- | --- | --- |
|  | **Unadjusted PR  (95% CI)** | **Adjusted ^d^ PR  (95% CI)** | **Unadjusted PR  (95% CI)** | **Adjusted ^d^ PR  (95% CI)** |
| Apathy | | | | |
| Rurality ^a^ |  |  |  |  |
| Rural | 0.93 (0.42-2.06) | 0.91 (0.37-2.23) | 0.71 (0.34-1.48) | 0.52 (0.22-1.19) |
| Small Urban | 0.65 (0.26-1.59) | 0.57 (0.21-1.55) | 0.51 (0.19-1.31) | 0.39 (0.15-1.06) |
| Large Urban | 1.00 ^e^ | 1.00 ^e^ | 1.00 ^e^ | 1.00 ^e^ |
| Median household income |  |  |  |  |
| Low (<$30,500) | 0.97 (0.41-2.26) | 1.32 (0.49-3.54) | 0.87 (0.39-1.95) | 1.77 (0.66-4.74) |
| Medium ($30,500-40) | 0.67 (0.28-1.63) | 0.75 (0.26-1.96) | 0.82 (0.38-1.75) | 1.66 (0.68-4.04) |
| High (>$40,000) | 1.00 ^e^ | 1.00 ^e^ | 1.00 ^e^ | 1.00 ^e^ |
| Residential instability ^b^ | 1.08 (0.84-1.16) | 1.01 (0.84-1.21) | 1.08 (0.76-1.04) | **0.81 (0.67-0.98) *** |
| Agitation | | | | |
| Rurality ^a^ |  |  |  |  |
| Rural | 0.78 (0.43-1.40) | 0.53 (0.27-1.05) | 0.76 (0.41-1.39) | 0.70 (0.344-1.43) |
| Small Urban | 0.76 (0.39-1.46) | 0.65 (0.31-1.39) | 0.49 (0.22-1.10) | 0.58 (0.25-1.34) |
| Large Urban | 1.00 ^e^ | 1.00 ^e^ | 1.00 ^e^ | 1.00 ^e^ |
| Median household income |  |  |  |  |
| Low (<$30,500) | 1.72 (0.92-3.22) | **2.42 (1.16-5.04) **** | 0.87 (0.45-1.68) | 1.21 (0.57-2.57) |
| Medium ($30,500-40) | 1.27 (0.66-2.43) | 1.73 (0.85-3.53) | 0.67 (0.36-1.27) | 0.93 (0.45-1.94) |
| High (>$40,000) | 1.00 ^e^ | 1.00 ^e^ | 1.00 ^e^ | 1.00 ^e^ |
| Residential instability ^b^ | 1.07 (0.86-1.14) | 0.98 (0.85-1.12) | **0.88 (0.77-0.99) *** | **0.86 (0.75-0.99) *** |
| Irritability | | | | |
| Rurality ^a^ |  |  |  |  |
| Rural | 0.97 (0.48-1.99) | 0.56 (0.24-1.29) | 0.82 (0.41-1.64) | 0.46 (0.24-1.05) |
| Small Urban | 0.46 (0.20-1.06) | **0.37 (0.14-0.98) *** | 0.35 (0.14-0.91) | 0.24 (0.09-0.62) |
| Large Urban | 1.00 ^e^ | 1.00 ^e^ | 1.00 ^e^ | 1.00 ^e^ |
| Median household income |  |  |  |  |
| Low (<$30,500) | 2.02 (0.93-4.40) | **2.98 (1.22-7.27) **** | 1.31 (0.61-2.80) | 2.08 (0.93-4.66) |
| Medium ($30,500-40) | 1.36 (0.61-3.06) | 2.06 (0.86-4.94) | 0.95 (0.46-1.97) | 1.47 (0.70-3.09) |
| High (>$40,000) | 1.00 ^e^ | 1.00 ^e^ | 1.00 ^e^ | 1.00 ^e^ |
| Residential instability ^b^ | 1.02 (0.86-1.19) | 0.95 (0.81-1.11) | 0.94 (0.82-1.09) | 0.93 (0.81-1.07) |

^a^ Rurality was measured based on the RUCA (Rural Urban Commuting Area codes) where large urban was defined as metropolitan area core; small urban was defined as metropolitan area high commuting and metropolitan area low commuting; and rural was defined as micropolitan area core, micropolitan high commuting, micropolitan low commuting, small town core, small town high commuting, small town low commuting, and rural areas.

^b^ Residential instability was defined as the percent who moved the past year.

^c^ Other race/ethnicity included non-Hispanic white (97.8%), Hispanic/Latinx (1.1%), and Asian (1.1%).

^d^ Model was adjusted for individual Alzheimer’s Disease patient age, sex/gender, and caregiver educational attainment.

^e^ Reference category.

^*^p<.05; ^**^p<.01

Bolded estimates indicate statistical significance.

# Supplemental Figure 1. Flow Chart of Study Eligibility among Individuals with Alzheimer’s Disease, Sample from South Carolina Alzheimer’s Disease Registry, 2010

**224** individuals with AD living at home with a caregiver

**12** excluded because missing geographic information (e.g., PO Boxes)

**605** analyzed participants from the sample collected from the SC Alzheimer’s Disease Registry, 2010

**352** individuals with AD admitted to nursing home excluded because mailing addresses were not collected

**59** individuals with AD living in the community alone excluded because mailing addresses were not collected

**Final Analytic Sample**

**212** co-habiting individuals with AD and caregivers accurately matched to US Geological Survey geocode using ArcGIS v10.2.1
